# The effects of inflammation and anti-inflammatory treatment on corneal endothelium in acute anterior uveitis

**Published:** 2019

**Authors:** Ana Cristina Ghiță, Larisa Ilie, Aurelian Mihai Ghiță

**Affiliations:** * Ocularcare Eye Clinic, Bucharest, Romania; ** Department of Ophthalmology, University Emergency Hospital, Bucharest, Romania; *** Department of Physiology II, “Carol Davila” University of Medicine and Pharmacy, Bucharest, Romania

**Keywords:** acute unilateral anterior uveitis, corneal endothelium, specular microscopy, local anti-inflammatory treatment

## Abstract

**Objective:** To establish the magnitude of endothelial cells alterations in acute anterior uveitis (AAU) and the clinical impact of local anti-inflammatory treatment.

**Methods:** 27 patients at first episode of unilateral AAU were included. According to the moment of presentation in our departments, two different groups were created, early treatment uveitis group (ETUG) and delayed treatment uveitis group (DTUG). Each patient underwent a corneal endothelial specular microscopy, in both eyes, two weeks after we begun the topical treatment.

**Results:** A statistically significant endothelial cells loss in the uveitis eye was identified in both groups, more important in DTUG. Also, in this group, the pleomorfism, polimegathism and central corneal thickness (CCT) were statistically significant increased.

**Conclusions:** The patients with unilateral AAU and delayed presentation showed more important alterations, first structural and then functional, due to a prolonged inflammatory response. This, in association with other favorable ocular conditions can progress to a permanent corneal endothelial decompensation. The sooner the anti-inflammatory treatment was initiated, the more limited were the destructive processes.

**Abbreviations:** AAU = Acute Anterior Uveitis, ETUG = Early Treatment Uveitis Group, DTUG = Delayed Treatment Uveitis Group, CCT = Central Corneal Thickness, CV = Coefficient of Variation, ECD = Endothelial Cells Density, HEX = Percentage of Hexagonal Cells

## Introduction

AAU is an acute inflammation of the ciliary body’s pars plicata and/or iris. The inflammatory response mainly involves the presence of Th1, Th17 lymphocytes and IL6, IL17, INF– γ, TNF-α cytokines which bring to the sites of interest other leukocytes from the circulation [**1**,**2**]. In AAU, the joining of cellular and humoral inflammatory factors directly affects the corneal endothelium [**3**] among other ocular structures. The anterior segment inflammation from AAU leads to structural changes, some of them being extremely dreadful, such as secondary glaucoma. In this research we want to analyze corneal endothelial changes in eyes with unilateral AAU and the behavior of the endothelium under topical anti-inflammatory treatment because, corneal endothelium is frequently neglected during and after a first episode of uveitis, excluding conditions such as uveitic glaucoma where endothelial damage occurs due to synechiae formation with iridocorneal contact and ocular hypertension [**4**,**5**]. In different studies, specular and confocal microscopy analyses confirm the presence of corneal endothelium changes revealed during anterior segment examination, such as numerous cornea guttata of different sizes and shapes, a large variety of keratic precipitates [**6**,**7**], endothelial pigment, and cellular debris. 

In the literature, there are still lots of different results which generate debates regarding corneal endothelial alterations after an acute anterior pole inflammation. [**3**,**8**-**10**]. 

## Methods

The study protocol was approved by the Ethics Committee of the University Emergency Hospital of Bucharest. A total number of 27 Caucasian patients with non-recurrent unilateral AAU participated to this study. According to their presentation at the ophthalmologist, two groups were formed. In ETUG we enrolled 12 patients who came to ask for medical care from the initial symptoms of disease and started local treatment. The second group, (DTUG), included 15 patients who searched for medical advice more than five days from the onset. Every patient with unilateral AAU underwent an endothelial analysis on both eyes with specular microscope Tomey EM-3000 after two weeks of local treatment, by the same investigator. Treatment included mydriatics, nonsteroidal and steroidal anti-inflammatory local eye drops, four times daily. The preferred method for data collection consisted in a succession of three automated images captured from the center of the endothelium. The mean data results were noted for each investigated parameter. The parameters of interest for both eyes, the eye with AAU and the congener eye were CV, ECD, the percentage of hexagonal cells (HEX) and CCT. From the research were excluded patients known with a history of intraocular surgery and other ocular diseases that could have generated corneal endothelium alterations. The collected data were studied with IBM SPSS Statistics 19. The general elements of descriptive statistics for graphic representation were used. Student’s t-test and One Way Anova Test were used. Statistically significant results were considered when p values were under 0.05 and shown as a mean +/- standard deviation.

## Results

There are slightly differences between the groups as regarding age and sex distribution. In ETUG the mean age is 44.27 years old with a female predominance (58.3%), while in DTUG is 38.06 years old with a male preponderance (60%). 

First of all, we compared the data between the eyes in ETUG and DTUG. In ETUG, ECD in the uveitis eye was smaller and statistically significant as compared to the congener eye 2.269 ± 405.83 cells/mm2 versus 2.359.17 ± 411.43 cells/mm2 (p<0.01). Also, we encountered an increased pleomorphism in the uveitis eye compared to the congener eye due to a drop in HEX of 53.417 ± 11.60 % versus 58.08 ± 16.56 %. The corneal endothelial cells polymegathism was similar between the eyes and therefore the values of 33.42 ± 9.7 and 33.75 ± 10.63 had no statistically significance. The CCT presented similarity in uveitis and congener eyes. (**Table 1**).

**Table 1:** Central corneal endothelial specular microscopy morphometric analysis results in ETUG

ETUG		Central Corneal Thickness (μm)	Average cell size (μm2)	Endothelial Cells Density (cells/ mm2)	Standard Deviation	Coefficient of Variation	Percentage of Hexagonal Cells (%)
Congener eye	Mean	551.58	438.83	2359.17	141.42	33.75	58.08
	Standard Deviation	49.17	115.98	411.43	51.38	10.63	16.56
Uveitis eye	Mean	544.92	458.92	2269.00	156.75	33.42	53.42
	Standard Deviation	59.65	115.35	405.83	53.60	9.70	11.60

In DTUG, the changes were even more important than in ETUG, with lower and statistically significant values (p<0.01) of the ECD in the uveitis eyes, of 2.541± 351.21 cells/ mm2 compared to the congener eyes, 2.792.13 ± 168.31 cells/ mm2. Both corneal endothelial pleomorphism and polymegathism, were statistically significant higher in the uveitis eyes p<0.01 than the congener eyes. HEX in uveitis eye is 51.33 ± 17.19%, lower than 67.73 ± 19.16% in the other eye. Also, we encountered a CV of 34.07 ± 7.05, higher than 29.67 ± 8.80 in the non-uveitis eye. In addition, an increase in CCT of 562.40 ± 51.28 μm was observed in the uveitis eye indicating a decrease in corneal endothelial pump activity compared with 538.27 ± 40.27 μm in the congener eye, p<0.05. (**Table 2**).

**Table 2:** Central corneal endothelial specular microscopy morphometric analysis results in DTUG

DTUG		Central Corneal Thickness (μm)	Average cell size (μm2)	Endothelial Cells Density (cells/ mm2)	Standard Deviation	Coefficient of Variation	Percentage of Hexagonal Cells (%)
Congener eye	Mean	538.27	359.40	2792.13	106.33	29.67	67.73
	Standard Deviation	40.27	22.93	168.31	35.22	8.80	19.16
Uveitis eye	Mean	562.40	403.80	2541.00	139.93	34.07	51.33
	Standard Deviation	51.28	73.38	351.21	45.11	7.05	17.19

Regarding the results from uveitis eyes and non-affected contralateral eyes between the two groups, we can observe that the differences are much higher in DTUG than in ETUG and also statistically significant.

## Discussion

In the literature are reported corneal endothelial cells alterations due to the anterior segment inflammatory response from an acute anterior uveitis. But, the duration between time of onset and presentation to the ophthalmologist and options of local treatment were not clearly defined. 

After two weeks of topical nonsteroidal and steroidal anti-inflammatory treatment, specular microscopy analysis of the uveitis eye showed different results in the studied groups. In DTUG the ECD presented a decrease in the eyes with uveitis than in congener eyes with 9.23 ± 9.44%. The drop was three times lower (3.74 ± 4.60%) as for ETUG uveitis eyes compared with contralateral eyes. HEX in uveitis eyes experienced a decrease of 19.95 ± 29.66% as reported to the non-affected eyes in DTUG compared with ETUG where the HEX was less with 3.09 ± 26.84% in the uveitis eyes than in congener eyes. The polymegathism was also greater and had statistically significance with 23.48 ± 39.57% in uveitis eyes as compared to the congener eyes in DTUG and with 0.20 ± 9.84% in ETUG (**Table 3**). 

**Table 3:** Percentage differences between the recorded parameters in congener and uveitis eyes in both study groups

Study Group		CCT difference congener-uveitis (%)	Average cell size difference congener-uveitic (%)	ECD difference congener-uveitis (%)	Standard deviation difference congener-uveitis (%)	CV difference congener-uveitis (%)	Hexagonal Cells difference congener-uveitis (%)
DTUG	Mean	4.50	12.01	9.23	40.62	23.48	19.95
	Standard Deviation	5.46	16.23	9.44	54.87	39.57	29.66
ETUG	Mean	-1.22	4.88	3.74	12.75	.20	3.09
	Standard Deviation	6.39	4.73	4.60	19.54	9.84	26.84

In ETUG, the values of polymegathism and pleomorphism are slightly different in the uveitis eye compared to the congener eye and are not statistically significant. The patients with unilateral AAU and delayed presentation showed more important structural and functional alterations, statistically significant, than those patients who searched for a prompt medical advice. Therefore, judging by the moment of treatment initiation we can say that inflammatory phenomena from acute anterior uveitis are able to generate endothelial dysfunction directly proportional to its intensity and duration. A slightly lower CCT in the uveitis eye than in the contralateral eye encountered in ETUG (**Table 1**) is a discordant result that can be partial clarified by the combination of both steroidal therapy effects, reduction of inflammation and corneal endothelial cells activity improvement combined with a potentiation of corneal endothelial’s pump function.

Sometimes, patients may present with recurrent AAU that can lead to significant endothelial changes in a cumulative manner. This, associated with other ocular conditions such as cataract surgery or other ocular disorders, can lead to a permanent endothelial decompensation, drop of visual function and quality of life. It was observed a better preservation of corneal endothelium under readily anti-inflammatory treatment initiation suggesting that inflammatory factors are directly responsible.

One limit of the research consists in a relatively low cohort generated from the restriction of inclusion criteria in order to obtain proper results. Many of the patients with AAU encountered in medical practice were already known with recurrent episodes in the same or in both eyes. Also, another limitation can be linked to the modality of data collection, as we examined only the central corneal endothelial morphology and not also the paracentral and peripheral corneal endothelium. Others researchers have shown that endothelial cells have an uneven distribution in the cornea. A 5.8% increase in the paracentral cornea and a 9.6% increase in the periphery were found, compared to the center of the cornea [**11**,**12**]. But of course, an extended study that includes not only central but also paracentral and peripheral endothelial measurements could provide more information about endothelial cells morphology. 

Thus, is important to continue the investigation of this patients treated from the beginning of the disease as well as those whose medical treatment was initiated later, to assess whether these corneal endothelium changes are permanent or reversible. 

## Conclusions

AAU causes not only specific slit-lamp changes such as keratic precipitates, multiple corneal guttae or corneal edema, but also other important morphological corneal endothelium changes like ECD loss, an increased pleomorphism and polymegathism, thus compromising endothelial and visual function. Early anti-inflammatory local treatment is essential because it can modulate the magnitude of the inflammatory phenomena from the anterior pole thus limiting the intensity and progression of endothelial alteration.

**Disclosures**

None.
